# Health economic evaluation alongside randomised clinical trial of a health behaviour intervention to manage type 2 diabetes in Nepal

**DOI:** 10.1186/s41256-024-00364-z

**Published:** 2024-12-17

**Authors:** Padam Kanta Dahal, Zanfina Ademi, Lal Rawal, Rashidul Alam Mahumud, Grish Paudel, Biraj Karmacharya, Haruka Sakamoto, Tomohiko Sugishita, Corneel Vandelanotte

**Affiliations:** 1 School of Health, Medical and Applied Sciences, Central Queensland University, Sydney Campus, Sydney, NSW Australia; 2 Appleton Institute, Physical Activity Research Group, Central Queensland University, Rockhampton, QLD Australia; 3 Health Economics and Policy Evaluation Research (HEPER) Group, Centre for Medicine Use and Safety, Faculty of Pharmacy and Pharmaceutical Sciences, Monash University, Melbourne, Australia; 4 School of Public Health and Preventive Medicine, Monash University, Melbourne, Australia; 5 Translational Health Research Institute (THRI), Western Sydney University, Sydney, NSW Australia; 6 NHRMC Clinical Trials Centre, Faculty of Medicine and Health, The University of Sydney, Camperdown, NSW Australia; 7 Department of Community Medicine, Kathmandu University Hospital, Dhulikhel, Nepal; 8 Section of Global Health, Department of Hygiene and Public Health, Tokyo Women’s Medical University, Tokyo, Japan

**Keywords:** Cost-effectiveness, Economic evaluation, Health behaviour interventions, Type 2 diabetes

## Abstract

**Background:**

Prevention of type 2 diabetes is becoming an urgent public health concern in low and middle-income countries (LMICs). However, there is currently no evidence of a cost-effective approach of health behaviour interventions from community settings in low-income countries like Nepal. Therefore, this study aimed to assess the within-trial economic evaluation of a health behaviour intervention compared with usual care for managing type 2 diabetes in a community setting in Nepal.

**Methods:**

We randomly assigned 30 clusters comprising 481 patients with type 2 diabetes of which 15 to a health behaviour intervention (*n* = 238 patients) and 15 to the usual care (*n* = 243 patients). Patients in the intervention group received community health workers-led intensive training for diabetes self-management along with regular phone calls and ongoing support from peer supporters. Costs, quality-adjusted life years (QALYs) and incremental cost-effectiveness ratio (ICER) as costs per QALYs gained were assessed after 6-month from a healthcare system perspective. Probabilistic sensitivity analysis was conducted using 10,000 Monte Carlo simulations to assess the impact of uncertainty of cost-effectiveness analysis under the threshold of three times gross domestic product (GDP) per capita for Nepal (i.e., US $4,140).

**Results:**

Over the 6-month, the intervention yielded an incremental cost of US $28.55 (95% CI = US $21.26 to US $35.84) per person and an incremental QALYs of 0.0085 (95% CI = -0.0106 to 0.0275) per person. The ICER associated with the health behaviour intervention was US $3,358.82 (95% CI = US $-2005.66 to US $3,974.54) per QALY gained, which was below the estimated threshold, indicating a cost-effective approach with a net monetary benefit of US $6.64 (95% CI = US $-22.62 to US $78.01). Furthermore, the probabilistic sensitivity analysis consisting of 10,000 Monte Carlo simulations indicates that the intervention being cost-effective at the given threshold was 89.63%.

**Conclusions:**

Health behaviour interventions in community settings are a cost-effective approach to manage type 2 diabetes, offering good value for money. However, more studies focused on long-term follow-up across diverse setting of LMICs should be warranted to assess the maximum impact of such interventions.

**Trial registration:**

Australia and New Zealand Clinical Trial Registry (ACTRN12621000531819) Registered on 6^th^ May 2021.

**Supplementary Information:**

The online version contains supplementary material available at 10.1186/s41256-024-00364-z.

## Background

The increasing prevalence of type 2 diabetes in lower-middle-income countries creates substantial burden in the healthcare system [1]. In Nepal, the prevalence of diabetes in 2022 was 8.5%, with a higher prevalence observed in male, older, urbanised, overweight, high blood pressure and high triglyceride populations [2]. This translates to a total healthcare expense of US $115.8 million in 2021 and this figure is expected to increase to US $190.5 million and US $168.1 per person by the year 2045 [3].

Health behaviour change interventions (e.g., diet, physical activity, foot care, medication intake) are effective, cost-effective and cost-saving for the care management of type 2 diabetes and its complications [4–6]. The trial-based cost-effectiveness analysis of health behaviour intervention in the Kerala Diabetes Prevention Program in India in 2020 showed that the incremental quality adjusted life years (QALYs) over 2 years was 0.04 per person and the incremental cost was US $50 per QALY gained [6]. The Indian Diabetes Prevention Program (IDPP) estimated a total cost of US $1,052 per case prevented over a 3-year time period [4]. Further, the modelling study of the Da Qing Diabetes Prevention Program in China over a 30-year time horizon predicted that health behaviour intervention was cost-saving and associated with better health outcomes (i.e., QALY increase of 0.74 per patient) [5]. In addition, a recent systematic review on economic evaluations of health behaviour interventions to manage type 2 diabetes in Asian countries demonstrated value for money, however, a majority of the included studies were from high-income community settings [7]. There is no direct evidence from low-income countries like Nepal. Furthermore, there are limited healthcare resources available and high out-of-pocket expenses for type 2 diabetes patients within the context of lower middle-income nations [8, 9].

Despite the Himalayan landscape and rich cultural heritage, Nepal is facing the rising burden of type 2 diabetes due to changing lifestyles, limited healthcare resources and infrastructures, and disparity in access to and utilisation of essential healthcare services [10]. Further, the high healthcare cost to manage type 2 diabetes has been a serious public health concern in the country. In this context, implementation of health behaviour intervention focusing on components of self-care practices such as dietary adherence, physical activities, healthcare utilisation, regular blood-glucose monitoring, oral health, footcare, diabetes medication adherence, and social and emotional support strategies are crucial for managing type 2 diabetes in Nepal [11]. Therefore, our cost-effectiveness analysis of a community-based health behaviour intervention through intensive training led by community health workers (CHWs), peer supporters and phone calls emerges as a crucial tool in resource allocation and decision-making processes in the management of type 2 diabetes. Further, our study plays a vital role in optimising health outcomes and reducing the economic burden of type 2 diabetes.

There is no evidence on the cost-effectiveness of health behaviour interventions that apply health education, regular phone calls and peer support to inform policy decisions regarding type 2 diabetes management in community settings in Nepal. Therefore, this study aimed to assess the within-trial economic evaluation of a health behaviour intervention compared with usual care for managing type 2 diabetes in a community setting in Nepal.

## Methods

### Study design, setting and participants

A within-trial cost-effectiveness analysis was performed alongside a cluster randomised control trial (Co-LID) with 6 months follow-up (April 2022 to September 2022), from a Nepalese healthcare system perspective. Participants were enrolled and randomised in the evaluation of a community-based health behaviour intervention among the patients living with type 2 diabetes. The Co-LID trial commenced in the Kavrepalanchok and Nuwakot districts of Nepal. Adults aged 30–70 years who were clinically diagnosed with type 2 diabetes were included in the study. Participants with type 1 or gestational diabetes or who were pregnant or not able to participate due to disability were excluded from this study.

Details of the trial design, intervention, participants and sample size have been described in detail elsewhere [11]. Briefly, we randomly assigned a total of 30 clusters comprising 481 patients with type 2 diabetes of which 15 to a health behaviour intervention group (*n* = 238) and 15 to control group (*n* = 243). The randomisation was conducted by the statistician not involved in this study using a computer generated random list to randomly assign (1:1) clusters to the intervention or control group.

### Intervention

The intervention was developed based on the importance of health behaviour in the prevention and management of type 2 diabetes, utilising the principle of the reach, effectiveness, adoption, implementation and maintenance (RE-AIM) framework [12, 13]. The intervention comprised of 12 modules of intensive face-to-face training sessions of self-management practices such as physical activity, dietary adherence, strategies to abstain from drinking and smoking, medication adherence, healthcare utilisation, blood sugar monitoring, footcare, complication reduction and social and emotional support, which were delivered fortnightly for 6 months by trained community health workers. Automated phone calls and text reminders were delivered to the participants for the reminder of the upcoming sessions. Additionally, the trained community health workers conducted fortnightly phone calls for the first 3 months followed by monthly to ensure continuing healthy self-care behaviour. This was supplemented by mobile phone messages on health behaviour change. Finally, peer supporters (i.e., two on each cluster of the intervention group) who were trained to work closely with the community health workers provided the necessary support to organise the group-based sessions. This included inviting the participants to the sessions, engaging and encouraging the participants to adopt self-care behaviour, and providing social and emotional support. Participants in the usual care group received standard care available at the local health facilities along with a pictorial book on diabetes prevention and management in Nepali.

### Measurement and valuation of healthcare use and costs

We considered medical consultation, screening, medication, hospitalisation, transportation, and consumption of recommended food items to manage and care for type 2 diabetes as a healthcare resource. The consultation visits with healthcare professionals such as cardiologists, dieticians, diabetes educators, podiatrists, ophthalmologists, endocrinologists, psychologists, physiotherapists, health assistants, nurses, community medicine assistants (CMA), and auxiliary nurse midwives (ANM) were considered as medical consultation. Diagnostic tests such as glycated haemoglobin (HbA1c), electrocardiogram (ECG), diabetic retinopathy, and footcare examination were considered screening resources. The uses of medicine to manage type 2 diabetes and its co-morbidities were under medication resources. Further, inpatient hospital visits including the number of admissions, overnight stays, and length of stays were identified as hospitalisation. The time to visit the nearest healthcare facilities was categorised as a travel time, and adherence to recommended fruits and vegetables consumption were identified.

Direct medical costs included were screening costs, intervention costs and health service utilisation costs (i.e., clinical visits, medications and hospitalisations) for type 2 diabetes up to 6 months follow-up. Screening frequency and health service utilisation data was obtained from participant reported data. The price of each service was estimated based on the health service price list of Dhulikhel Hospital, which is located in a semi-urban community of Kavrepalanchok district in Nepal. This hospital operates several outreach health centres in the Kavrepalanchok and Nuwakot districts, which are heavily relied upon by the patients of our study sites. Furthermore, we assessed health expenditures for both outpatient and inpatient care, encompassing private and public healthcare centres, out-of-pocket expenses, and health insurance charges, all within a 6-month duration as part of the health service utilisation analysis. In the intervention, the costs for health educators, peer support, volunteers (e.g., female community health volunteers (FCHVs)), health workers, phone calls and short message service (SMS) costs and materials costs were estimated based on the number of services provided, the total number of visits and phone calls to the patients. The costs of intervention sessions, including participant attendance, daily salary rates for health workers, and hypothetical payments for volunteers and peer support, were estimated. Phone and message costs were calculated based on the number of calls and messages delivered to the patients in the intervention group, taking into account the average charges per minute and per message from Nepal Telecommunication Authority [14]. Material costs for the interventions were collected from the project management team.

Direct non-medical costs included the expenditure for the participants transportation, food and vegetable consumption, accommodation while seeking health care services, waiting time and time spent travelling to sessions or receiving phone advice. The transportation time to each facility or session was obtained from the Government of Nepal, Ministry of Physical Infrastructure and Transport, Department of Transport Management [15]. Food and vegetable prices were obtained from local market prices per serving per day during the time of data collection [16]. Servings of fruits and vegetables followed the American Heart Association guidelines and infographic, defining one serving of fruit as a medium-sized fruit (e.g., an apple) and considering a cup of salad or half a cup of cooked vegetables as one serving [17].

Indirect costs were calculated based on participants’ productivity losses, assuming an 8-h workday for inpatient hospital stays and a 4-h workday for outpatient visits, this is based in the human capital approach. Time was valued at the minimum unskilled labour wage set by the Nepal Government, given the agriculture nature of the community-based intervention [18].

The costs were estimated in accordance with the Consolidated Health Economic Evaluation Reporting Standards (CHEERS) statement [19] and guidelines for economic evaluations (Supplementary Table 1) [20]. Costs for the purpose of the research itself, such as data collection, and analysis were excluded from the analysis. Further, costs were calculated in Nepali rupees and converted to US dollars based on the average exchange rate of 2022 (i.e., US $1 = NRs 125.20) [21].

### Outocme selection, measurement and valuation

Effectiveness of the intervention was assessed in terms of QALYs (i.e., year of life spend × utility score). We assessed utility-based quality of life (QoL) using scores from the 3-level EQ-5D version (EQ-5D-3L) instrument that assesses preference-based health related quality of life [22]. It has five dimensions that include mobility, self-care, usual activities, pain/discomfort, and anxiety/depression with 3 probable responses (no problems, some problems, and extreme problems). The Time Trade Off (TTO) method was used to assign a utility score to each of the EQ-5D health states. The TTO method is a choice-based method of assessing a health utility state that reflects the length of life expectancy received by each person. The EQ-VAS score was measured from 0 to 100 which is the worst and best health respectively that the participant imagines, where 1 is considered as perfect health and 0 is death [23]. The mean and standard deviation of the VAS score was reported to describe the overall health of the patient at the day of data collection. A Nepal specific algorithm of the EQ-5D-3L does not exist, therefore, we applied the Indian population tariff to calculate the utilities where reverse crosswalk mapping function was applied [24].

### Cost-effectiveness analysis

The main outcome for this economic evaluation was the incremental cost-effectiveness ratio (ICER) in terms of cost per QALY gained [25].

An ICER was calculated as the total cost for the intervention minus the total cost for the control divided by the total QALYs of the intervention minus the QALYs of the control:

1 $$\text{ICER}= \frac{{\text{Intervention}}_{\text{costs}}- {\text{Control}}_{\text{costs}}}{{\text{Intervention}}_{\text{QALYs}}-{\text{Control}}_{\text{QALYs}}}$$

These findings were illustrated using a cost-effectiveness plane, along with the associated probabilities of being cost-effective and cost saving.

The cost-effectiveness threshold of Nepal was calculated based on the World Health Organisation Choosing Interventions that are Cost-effective (WHO-CHOICE) project recommendations, i.e., ICER less than three times national gross domestic product (GDP) per capita [25]. According to the international monetary fund (IMF), the GDP per capita for Nepal in August 2023 was US $1,380, therefore we considered US $4,140 as a threshold [26]. As the intervention period is less than 1 year, the discounting rate was not applied to calculate costs and QALYs [27, 28].

### Statistical and sensitivity analyses

An intention-to-treat analysis approach was applied for the analysis given that the economic evaluation was guided by the standard methods of within-trial economic evaluation. Data related to costs and QALYs were highly skweded, making statistical significance differences using the parametric approach was infeasible. Data were reported as means and standard deviations, and mean differences between intervention and control groups with 95% CIs. Based on the nature of the data characteristics and model assumptions, a bootstrap with generalised linear model (GLM) (with Gamma family and identity link function for costs as a dependent variable and with Gaussian family distribution with identity link function for QALYs as a dependent variable) was used to assess the statistical significance differences with 95% CIs in costs and QALYs. We applied ‘modified park test’ to determine the appropriate family distribution for a GLM by testing the relationship between the mean and the variance of the dependent variable [29]. Furthermore, we used a bootstrapping approach with 10,000 replications to calculate 95% CIs around mean total costs and mean total effects.

In the GLM, costs and QALYs were considered as the dependent variable, where age and gender were adjusted for costs and baseline utility was adjusted for QALYs. The following GLM model mathematical equation was applied.

2 $$y={\beta }_{0}+{{\beta }_{1}{\delta }_{i}+\beta }_{2}{x}_{1}+{\beta }_{3}{x}_{2}+\dots + \epsilon$$

where the index i is the patient identifier (i = 1, 2, …, N), ${\delta }_{i}$ is a treatment arm dummy variable (0 = control; 1 = intervention), *y* is the dependent variable (i.e., costs or QALYs), ${\beta }_{1}$ represents the adjusted differential costs or QALYs after controlling for factors (${x}_{\text{i}})$. Further, ICER was estimated by diving incremental costs with the incremental QALYs and the Net Monetary Benefit (NMB) was calculated as the difference between the incremental benefit multiplied by threshold value and the incremental cost (i.e., NMB = (Incremental benefit × threshold value) – Incremental cost) [30]. A positive NMB suggests that the health behaviour intervention is expected to generate more value than its costs, indicating its cost-effectiveness. However, negative implies its value is less than the additional cost of the benefits, suggesting that the interventions may not be cost-effective [31].

In the base case analysis, the incremental costs and QALYs gained over the 6 months of health behaviour interventions were calculated. A sub-group analysis was conducted by stratifying the costs and effects based on patients’ residency status (i.e., distinguishing between semi-urban and rural), which helped to explore the cost-effectiveness across diverse settings. Further, we systematically excluded cost of each parameter (i.e., phone call, peer support and intensive training intervention) at least once, generating ICERs for each exclusion. In addition, as a scenario analyses, the two main costing parameters; the intervention costs and outcomes were increased and decreased by 10% in both arms and the ICERs were subsequently generated. This helped to address the challenges faced by cost fluctuation, as well as the potential for underestimation and overestimation of healthcare resource used and provide a more contextual study.

A probabilistic sensitivity analysis was conducted using 10,000 Monte Carlo simulations which helped to estimate the impact of uncertainty of the cost-effectiveness analysis. The incremental costs and QALYs were used to generate a cost-effectiveness plane and the probability of cost-effectiveness and cost savings. Also, multiple imputation analyses were conducted to address the issues of missing data, involving the creation of five imputed datasets. Pooled values, derived from these datasets were used for data analyses by using Rubin’s rule [32]. Data cleaning, coding and all probabilistic sensitivity analyses were conducted in Microsoft excel (Version 16.77 (23091003)), data analyses were conducted in SPSS (Version 29.0.1.0 (171) and STATA/BE.18.0.

## Results

### Participants characteristics

The general characteristics of the participants in both groups are presented in the Table 1. Among the 481 participants, 92.10% (i.e., 443 (225 intervention and 218 control)) were followed up 6 months after the trial, whereas 7.90% (i.e., 38) of the participants were lost to follow up (See supplementary Figure 1, CONSORT diagram).

**Table 1:** Baseline characteristics of participants

**Characteristics**	**Intervention (*n* = 238)**	**Control (*n* = 243)**	***P*-value**
	Frequency (%)	Frequency (%)	
Age, Mean (SD)	54.26 (9.12)	54.62 (9.71)	0.675
Sex			
Male	120 (50.42)	134 (55.14)	0.300
Female	118 (49.58)	109 (44.86)	
Marital Status			
Married	221 (92.86)	226 (93.00)	0.950
Others (unmarried, divorced & widowed)	17 (7.14)	17 (7.00)	
Religion			
Hindu	210 (88.24)	219 (90.12)	0.469
Buddhist	20 (8.40)	18 (7.41)	
Christian	8 (3.36)	6 (2.47)	
Ethnicity			
Brahmin	87 (36.56)	100 (41.15)	0.483
Newar	70 (29.41)	45 (18.52)	
Chhetri	26 (10.92)	27 (11.11)	
Others (Janajati, Madhesi & Dalit)	55 (23.11)	71 (29.22)	
Education			
Literate	151 (63.45)	154 (63.37)	0.987
Illiterate	87 (36.55)	89 (36.63)	
Occupation			
Agriculture	91 (38.23)	95 (39.09)	0.495
Household activities	59 (24.79)	47 (19.34)	
Business	34 (14.29)	39 (16.05)	
Animal husbandry	10 (4.20)	10 (4.12)	
Others (Laborer, Retired Service, & Driver)	44 (18.49)	52 (22.40)	
Residency			
Semi-urban	153 (64.29)	156 (64.20)	0.984
Rural	85 (35.71)	87 (35.80)	
Utility, Mean (SD)	0.90 (0.14)	0.91 (0.12)	0.888

*P*-value was obtained from two sample independent t-test or chi-square test or Fisher’s exact test where appropriate

*n* Number of participants, *SD* Standard deviation, *%* Percentage

### Healthcare costs

The volume of resource use and their cost in Nepali Rupees (NRs) are presented in Supplementary Tables 2 and 3, with the costs associated with healthcare resources use and the health behaviour intervention throughout the 6-month trial period are presented in Table 2. The main cost driver was screening in both groups (i.e., 23% in intervention and 39% in control) (Supplementary Figure 2). However, healthcare resource use costs in terms of transportation and recommended food items across the intervention group were lower by 3% and 21% respectively compared to the control group. The average total cost of an intervention program for managing type 2 diabetes was US $17.33 (SD = 8.60) per patient, with the majority of expenses being driven by the intensive training led by the community health workers (Mean = US $14.33; SD = 7.27) followed by the peer supportters (Mean = US $2.53; SD = 1.37) and the phone calls (Mean = US $0.47; SD = 0.08). Total cost of healthcare resource use per patient over the 6-month trial in the intervention group (Mean = US $63.03; SD = 59.57) were higher by US $28.55 (95% CI = US $21.26 to US $35.84) compared to the control group (Mean = US $34.23; SD = 17.08).

**Table 2:** Estimated cost (US $) per patient throughout the 6 months trial

**Measurements**	**Intervention (*n* = 238)**		**Control (*n* = 243)**		**Mean differences (95% CI)**
	Mean	SD	Mean	SD	
Direct medical costs					
Medical consultation	4.16	6.28	3.85	7.05	0.31 (-0.87 to 1.49)
Screening	14.31	6.12	13.43	5.88	0.89 (-0.19 to 1.97)
Medication	9.35	17.73	3.68	8.40	5.66 (3.23 to 8.10)
Inpatient services	2.80	30.47	1.45	4.68	1.97 (-1.95 to 5.88)
Sub-total	30.62	60.60	22.41	26.01	8.83 (3.82 to 13.84)
Direct non-medical costs					
Transportation	0.64	0.77	0.66	0.68	-0.02 (-0.15 to 0.11)
Recommended food items	1.62	2.18	2.06	2.11	-0.43 (-0.80 to -0.05)
Sub-total	2.27	2.95	2.71	2.79	-0.45 (-0.84 to -0.05)
Indirect costs					
Patient income loss	12.83	24.88	9.11	6.55	3.81 (0.55 to 7.06)
Intervention cost					
Phone call	0.47	0.08	na	na	na
Peer support	2.53	1.37	na	na	na
Intensive training	14.33	7.27	na	na	na
Total intervention cost	17.33	8.60	na	na	na
Total costs	63.03	59.57	34.23	17.08	28.80 (20.98 to 36.62)

CIs were obtained from 10,000 bootstrap resampling technique using generalised linear model (GLM)

*na* Not applicable, *CI* Confident intervals, *SD* Standard deviation

### Health outcomes

Over the 6-month follow-up period, the average QALY for the intervention group and control was 0.4302 (SD = 0.1075) and 0.4227 (SD = 0.1052) respectively. This resulted in a QALY gain of by 0.0085 (95% CI = -0.0106 to 0.0275) compared to the control group (Table 3).

**Table 3:** Cost-effectiveness analysis throughout 6 months trial

**Outcomes**	**Unadjusted**		**Adjusted**	
	Intervention	Control	Intervention	Control
Total cost, Mean (SD)	US $63.03 (US $59.57)	US $34.23 (US $17.08)	US $63.03 (US $59.57)	US $34.23 (US $17.08)
QALY, Mean (SD)	0.4302 (0.1075)	0.4227 (0.1052)	0.4302 (0.1075)	0.4227 (0.1052)
Incremental cost, Mean (95% CI)	US $28.80 (US $20.98 to US $36.62)		US $28.55 (US $21.26 to US $35.84)^b^	
Incremental QALY gained Mean (95% CI)	0.0075 (-0.0116 to 0.0267)		0.0085 (-0.0106 to 0.0275)^c^	
ICER (cost per QALY gained), Mean (95% CI)	US $3,840.00 (US $-1,808.62 to US $4,005.23)		US $3,358.82 (US $-2005.66 to US $3,974.54)	
Cost-effectiveness^a^	Health behaviour intervention more costly & more effective			
Net monetary benefit, Mean (95% CI)	US $2.25 (US $-27.04 to US $73.91)		US $6.64 (US $-22.62 to US $78.01)	

CIs were obtained from 10,000 bootstrap resampling technique

*QALY* Quality adjusted life year, which was reported in four decimal points for higher precision in measurement, *ICER* Incremental cost-effectiveness ratio

^a^Decision was made based on the threshold value of 3-times GPD per capita of Nepal which is US $ 4,140 (US $1380 × 3) based on international monetary fund database on July 2023; Net monetary benefit was calculated as ((Incremental benefit × Threshold) – Incremental cost); ^b^adjusted age and gender, ^c^adjusted baseline utility value

### Cost-effectiveness and sensitivity analyses

The results of the cost-effectiveness analyses are presented in Table 3. The adjusted incremental cost-effectiveness ratio was US $3,358.82 (95% CI = US $-2005.66 to US $3,974.54) per QALY gained. The health behaviour intervention was proven to be cost-effective at the threshold of US $4,140. Further, the health behaviour intervention gained US $6.64 (95% CI = US $-22.62 to US $78.01) net monetary benefit over the course of the 6-month trial.

**Fig. 1:**
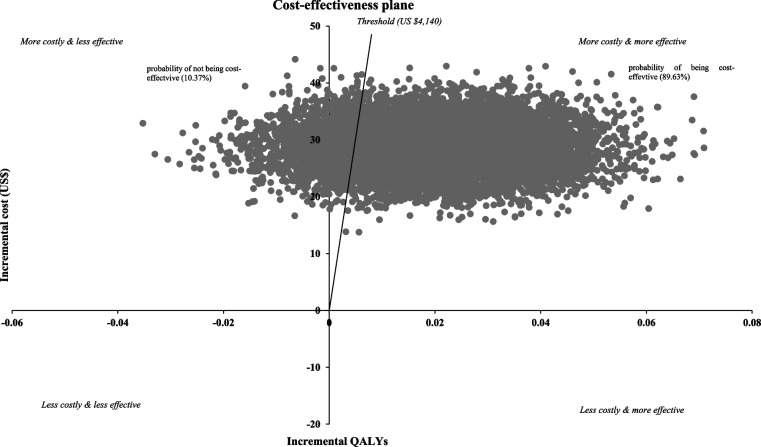
Cost-effectiveness plane by using 10,000 Monte-Carlo simulation throughout 6-months trial

The results of sub-group and scenario analyses are presented in Table 4. In the sub-group analysis, the ICER for semi-urban areas was US $1,253.19 per QALY gained, whereas for rural areas it was US $2,916.79 per QALY gained; thereby establishing the cost-effectiveness of the behaviour intervention in both settings. Scenario analyses demonstrated that the ICERs remained below the given threshold level, indicating the cost-effectiveness of the health behaviour intervention. For instance, excluding phone calls, peer support and intensive training interventions, resulted in ICER values of US $3,777.33 per QALY gained, US $3,502.67 per QALY gained, and US $1,929.33 per QALY gained, respectively. Further, when intervention costs and effects simultaneously increased and decreased, the ICER values were US $3,816.87 and US $3,811.76 per QALY gained, respectively. Probabilistic sensitivity analyses, consisting of 10,000 Monte Carlo simulations, revealed that the health behaviour intervention group had an 89.63% probability of being cost-effective compared to the control group, given a willingness to pay the threshold of US $4,140 (Fig. 1).

**Table 4:** ICER estimation based on the sub-group and scenario analyses

**Parameter**	**Intervention**		**Control**		**Mean incremental cost (95% CI)**	**Mean incremental QALY (95% CI)**	**ICER, US $ per QALY gained**
	Mean cost in US$	Mean QALY gained	Mean cost in US $	Mean QALY gained			
Residential status							
Semi-urban	60.23	0.4391	36.67	0.4203	23.56 (18.38 to 28.74)	0.0188 (-0.0024 to 0.0404)	1,253.19^a^
Rural	68.06	0.4273	29.85	0.4142	38.21 (18.38 to 58.04)	0.0131 (-0.0492 to 0.0232)	2,916.79^a^
Interventions							
Exclusion of phone call intervention	62.56	0.4302	34.23	0.4227	28.33 (20.51 to 36.15)	0.0075 (-0.0116 to 0.0267)	3,777.33^a^
Exclusion of peer support intervention	60.50	0.4302	34.23	0.4227	26.27 (18.46 to 34.08)	0.0075 (-0.0116 to 0.0267)	3,502.67^a^
Exclusion of intensive training intervention	48.70	0.4302	34.23	0.4227	14.47 (6.68 to 22.27)	0.0075 (-0.0116 to 0.0267)	1,929.33^a^
Scenario-1							
Intervention costs & effects increased 10%	69.33	0.4733	37.65	0.4650	31.68 (23.08 to 40.28)	0.0083 (-0.0127 to 0.0293)	3,816.87^a^
Intervention costs & effects reduced 10%	56.72	0.3869	30.81	0.3801	25.92 (18.88 to 32.96)	0.0068 (-0.0104 to 0.0239)	3,811.76^a^
Scenario-2							
Costs of screening & intensive training increased by 10%	65.89	0.4302	34.86	0.4227	31.03 (23.19 to 38.87)	0.0075 (-0.0116 to 0.0267)	4,137.33^a^
Costs of screening & intensive training reduced by 10%	60.16	0.4302	32.17	0.4227	27.99 (20.23 to 35.75)	0.0075 (-0.0116 to 0.0267)	3,732.00^a^

Decision was made based on the threshold value of 3-time GPD per capita of Nepal which is US $ 4,140 (US $1380 × 3)

CIs were obtained from 10,000 bootstrap resampling technique

*QALY* Quality adjusted life year, which was reported in four decimal points for higher precision in measurement, *ICER* Incremental cost-effectiveness ratio

^a^Health behaviour intervention is cost-effective

## Discussion

Type 2 diabetes is highly prevalent in low- and middle-income countries like Nepal, yet there are no economic evaluations of health behaviour interventions. To our knowledge, this is the first randomised controlled trial (RCT) to provide evidence on the economic evaluation of a health behaviours intervention, including phone calls, peer support and intensive training, for managing and caring for type 2 diabetes in Nepali community settings. Our study observed that, over the 6-month trial period, the intervention group incurred higher costs and produced greater effects in terms of QALYs compared to control group. Further, our results suggest that the health behaviour intervention was cost-effective and has potential to remain cost-effective across various subgroup analysis, resulting in a net monetary benefit of US $6.64 over the course of 6-month trial period, though it is noteworthy that the negative lower CI indicates the potential uncertainty of being cost-effectiveness.

Our findings indicate that the healthcare resource utilisation costs without intervention costs were higher by 33.51% (i.e., US $11.47) in the intervention group compared to control group, aligning with our study’s objective of increasing healthcare utilisation. Furthermore, upon implementing the 6-month trial, the costs raised by almost 83%, which can be attributed to the use of additional resources required to facilitate the adoption of health behaviour change. Notably, the primary cost drivers in our analysis were screening, medication, medical consultations, and the intensive training that was part of the intervention. A similar trend was observed in the Indian Diabetes Prevention Program (IDPP) study conducted by Ramachandran et. al., in 2007, where the first-year costs in the intervention group were higher due to screening, and phone calls for reviewing screening outcomes and providing motivation [4]. A similar scenario was also evident in the Kerala Diabetes Prevention Program (K-DPP) conducted in India in 2020, where the per-patient cost was US $24.2 in the intervention group and US $0.8 in control group [6]. Interestingly, healthcare resources utilisation costs were higher in the first year and decreased significantly in subsequent years [33]. This suggests a positive impact on the health of participants produced by the behaviour change intervention and better use of healthcare resources.

Our study indicated an improvement of 0.0085 QALYs (per patient) among the type 2 diabetes patients in the intervention group, which is almost 2% increase compared to control group. These results are consistent with other studies, including recent systematic reviews, indicating that health behaviour interventions were associated with a QALY gain of 0.01 to 0.14 per patient [7, 34]. However, due to the short-term follow-up of our study, the increased QALYs percentage is lower compared to previous health behaviour interventions, as there was less sufficient time to realise the full impact of behaviour change and their measurable benefits. The positive intervention effects could be further increased through implementing additional self-care support strategies, such as empowerment, psychosocial support and long-term planning [35].

Over the 6-month trial period, the current study identified that the health behaviour intervention was cost-effective, with approximately 20% net monetary benefits, considering the cost of usual care as a reference point. Importantly, this key finding was consistent across the sub-groups analyses, indicating a nine-fold probability of being cost-effective compared to the probability of being cost-saving. However, there is an uncertainty about the lower range of NMB, which is negative. Thus, decision-makers should carefully consider the uncertainty surrounding the cost-effectiveness of the intervention. This primarily due to the challenges associated with capturing short-term cost savings, as upfront costs in health behaviour changes can outweigh the immediate savings [36]. These findings align with previous systematic reviews within the Asian context, highlighting that health behaviour interventions for managing and caring for type 2 diabetes are cost-effective [7]. This review also indicated that health behaviour interventions become more cost-saving when the time horizon expands. For instance, the ‘Da Qing Diabetes prevention program’ in China demonstrated cost-savings of ¥5, 338 (i.e., approx. US $789 based on 0.1478 average exchange rate of 2022) per patient over 30 years and ¥1,921(US $284) for a lifetime time horizon [5]. A similar systematic review of economic evaluation studies examining the impact of physical activity interventions to manage type 2 diabetes in affluent nations found that these interventions were cost-effective for 60% of included studies and cost-saving for 40% of included studies [37]. These findings collectively emphasize that health behaviour interventions for managing type 2 diabetes are not only cost-effective, but ultimately lead to cost savings over a longer time horizon.

Our study findings have significant implications for clinical practice, healthcare policy formulation, and future research in Nepal. Firstly, understanding the cost-effectiveness of the intervention can better inform healthcare providers and clinicians about preventing and delaying the diabetes-related complications, reducing hospitalisations, and minimising medications costs. Furthermore, these results can empower healthcare decision-makers to allocate resources more efficiently towards preventive care measures and the development of diabetes prevention strategies. Ultimately, our study highlights the need for more research in low- and middle-income nations like Nepal, with long-term follow-up, to explore the sustained impact and cost-saving potential of health behaviour interventions.

Our study has several strengths. Within the Nepalese healthcare system context, this intervention was highly innovative, and it provides new evidence for decision-makers across various community settings. This study highlighted the importance of preventive and promotive strategies in controlling type 2 diabetes, which may have broad socio-economic benefits. This intervention can lead to more sustainable healthcare practices in low-resource community settings and equip patients with skills for healthier longer-term choices. However, this study has some limitations. Primarily, our RCT study relies on short-term follow-up with a sample limited to two specific geographical locations in Nepal, potentially making it challenging to observe significant behaviour changes that may not be generalisable to the entire nation. Moreover, our study depended on limited resources, which may impact the program’s ability to provide continuous support and feedback in the longer term. Further, our estimates are based on self-reported patient data, introducing potential reporting bias. Moreover, the cost of resources may change over the time, leading to over or underestimations of the healthcare costs. Lastly, our utility values, were based on India’s’ EQ-5D-3L algorithm, may introduce bias and the assumptions may not be directly applicable to Nepali context.

## Conclusions

Health behaviour changes interventions to manage type 2 diabetes that include intensive training on behaviour change along with regular phone calls and peer support in community settings can be a cost-effective approach to improve the health and well-being of people with type 2 diabetes. The evaluated intervention yielded higher monetary benefits and demonstrate progressively increasing effects. However, more studies with longer term follow-up are recommended to fully uncover the potential for cost savings. Similarly, future research should be warranted to implement across diverse settings of LMICs including Nepal to assess the long-term cost-effectiveness and sustainability of health behaviour change interventions. Such research is essential for informing evidence-based policy decisions and ensuring the continued efficacy of interventions aimed at combating T2DM and improving health outcomes.

## Supplementary Information

Supplementary Material 1.

## Data Availability

Data will be available upon the reasonable request to the corresponding authors.

## Abbreviations

T2D: Type 2 diabetes
LMICs: Low and middle-income countries
QALYs: Quality adjusted life years
IDPP: Indian Diabetes Prevention Program
RE-AIM: Reach, effectiveness, adoption, implementation and maintenance
CHEERS: Consolidated health economic evaluation reporting standards
TTO: Time trade off
WHO-CHOICE: World health organisation choosing interventions that are cost-effective
IMF: International monetary fund
GDP: Gross domestic product
ICER: Incremental cost-effectiveness ratio
NMB: Net Monetary Benefit
