# Supplementary material for: Health economic evaluation alongside randomised clinical trial of a health behaviour intervention to manage type 2 diabetes in Nepal
Source: Glob Health Res Policy. 2024 Dec 17;9:52. doi: 10.1186/s41256-024-00364-z (PMC11650842; doi:10.1186/s41256-024-00364-z)
Supplement: Supplementary file 1 — Supplementary Material 1. [file 41256_2024_364_MOESM1_ESM.docx]

**Supplementary files**

**Supplementary table 1: Consolidated Health Economic Evaluation Reporting Standards (CHEERS) statement checklists**

| **Topic** | **No.** | **Item** | **Location where item is reported** |
| --- | --- | --- | --- |
| Title |  |  |  |
|  | 1 | Identify the study as an economic evaluation and specify the interventions being compared. | Page 1 |
| Abstract |  |  |  |
|  | 2 | Provide a structured summary that highlights context, key methods, results, and alternative analyses. | Page 2-3 |
| Introduction |  |  |  |
| Background and objectives | 3 | Give the context for the study, the study question, and its practical relevance for decision making in policy or practice. | Page 3-5 |
| Methods |  |  |  |
| Health economic analysis plan | 4 | Indicate whether a health economic analysis plan was developed and where available. | Page 5-7 |
| Study population | 5 | Describe characteristics of the study population (such as age range, demographics, socioeconomic, or clinical characteristics). | Page-5 |
| Setting and location | 6 | Provide relevant contextual information that may influence findings. | Page 5 |
| Comparators | 7 | Describe the interventions or strategies being compared and why chosen. | Page 5 |
| Perspective | 8 | State the perspective(s) adopted by the study and why chosen. | Page 5 |
| Time horizon | 9 | State the time horizon for the study and why appropriate. | Page 5 |
| Discount rate | 10 | Report the discount rate(s) and reason chosen. | Page 10 |
| Selection of outcomes | 11 | Describe what outcomes were used as the measure(s) of benefit(s) and harm(s). | Page 6-9 |
| Measurement of outcomes | 12 | Describe how outcomes used to capture benefit(s) and harm(s) were measured. | Page 6-10 |
| Valuation of outcomes | 13 | Describe the population and methods used to measure and value outcomes. | Page 6-8 |
| Measurement and valuation of resources and costs | 14 | Describe how costs were valued. | Page 6-8 |
| Currency, price date, and conversion | 15 | Report the dates of the estimated resource quantities and unit costs, plus the currency and year of conversion. | Page 8 |
| Rationale and description of model | 16 | If modelling is used, describe in detail and why used. Report if the model is publicly available and where it can be accessed. | NA |
| Analytics and assumptions | 17 | Describe any methods for analysing or statistically transforming data, any extrapolation methods, and approaches for validating any model used. | Page 9-11 |
| Characterising heterogeneity | 18 | Describe any methods used for estimating how the results of the study vary for subgroups. | Page 10-11 |
| Characterising distributional effects | 19 | Describe how impacts are distributed across different individuals or adjustments made to reflect priority populations. | Page 10-11 |
| Characterising uncertainty | 20 | Describe methods to characterise any sources of uncertainty in the analysis. | Page 10-11 |
| Approach to engagement with patients and others affected by the study | 21 | Describe any approaches to engage patients or service recipients, the general public, communities, or stakeholders (such as clinicians or payers) in the design of the study. | NA |
| Results |  |  |  |
| Study parameters | 22 | Report all analytic inputs (such as values, ranges, references) including uncertainty or distributional assumptions. | Page 11 |
| Summary of main results | 23 | Report the mean values for the main categories of costs and outcomes of interest and summarise them in the most appropriate overall measure. | Page 12-16 |
| Effect of uncertainty | 24 | Describe how uncertainty about analytic judgments, inputs, or projections affect findings. Report the effect of choice of discount rate and time horizon, if applicable. | Page 13-16 |
| Effect of engagement with patients and others affected by the study | 25 | Report on any difference patient/service recipient, general public, community, or stakeholder involvement made to the approach or findings of the study | Page 13-16 |
| Discussion |  |  |  |
| Study findings, limitations, generalisability, and current knowledge | 26 | Report key findings, limitations, ethical or equity considerations not captured, and how these could affect patients, policy, or practice. | Page 16-19 |
| Other relevant information |  |  |  |
| Source of funding | 27 | Describe how the study was funded and any role of the funder in the identification, design, conduct, and reporting of the analysis | Page 21 |
| Conflicts of interest | 28 | Report authors conflicts of interest according to journal or International Committee of Medical Journal Editors requirements. | Page 21 |

*From: Husereau D, Drummond M, Augustovski F, et al. Consolidated Health Economic Evaluation Reporting Standards 2022 (CHEERS 2022) Explanation and Elaboration: A Report of the ISPOR CHEERS II Good Practices Task Force. Value Health 2022;25.* [*doi:10.1016/j.jval.2021.10.008*](doi:10.1016/j.jval.2021.10.008)


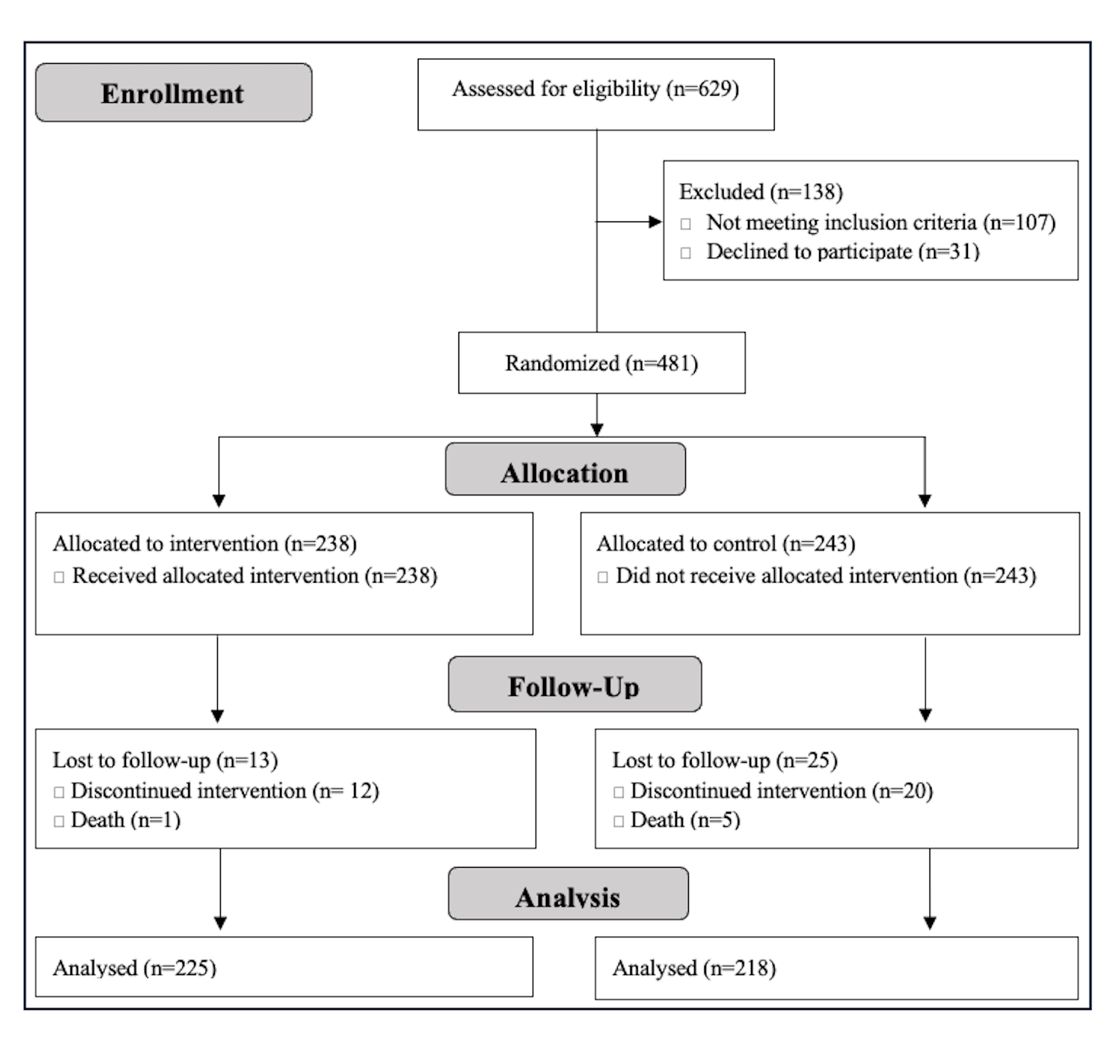


**Supplementary figure 1: CONSORT flow diagram for RCT**

**Supplementary table 2: Volume of resource use per patient throughout the 6 months trial**

| **Resources** | **Items** | **Intervention (n=225)** | **Control (n=218)** | **Mean difference (95% CI)** |
| --- | --- | --- | --- | --- |
|  |  | Mean (SD) | Mean (SD) |  |
| Medical consultation | Cardiologist | 0.22 (0.42) | 0.16 (0.37) | 0.06 (-0.06 to 0.17) |
|  | Dentist | 0.21 (0.41) | 0.05 (0.23) | 0.16 (0.07 to 0.25) |
|  | Dietician | 0.03 (0.16) | 0.00 (0.00) | 0.03 (-0.004 to 0.06) |
|  | Diabetes educator | 0.33 (0.47) | 0.43 (0.50) | -0.11 (-0.25 to 0.04) |
|  | Podiatrist | 0.01 (0.09) | 0.01 (0.12) | -0.01 (-0.04 to 0.03) |
|  | Ophthalmologist | 0.27 (0.44) | 0.30 (0.46) | -0.03 (-0.17 to 0.10) |
|  | Endocrinologist | 0.23 (0.42) | 0.18 (0.38) | 0.05 (-0.07 to 0.18) |
|  | Physiotherapist | 0.04 (0.19) | 0.04 (0.20) | -0.01 (-0.06 to 0.05) |
|  | Psychologist | 0.03 (0.16) | 0.03 (0.16) | 0.00 (-0.05 to 0.05) |
|  | Others (HA, Nurse, CMA, ANM) | 0.08 (0.03) | 0.12 (0.33) | -0.04 (-0.13 to 0.05) |
|  | Sub-total | 1.45 (2.80) | 1.32 (2.75) | 0.10 (-0.76 to 0.98) |
| Screening | HbA1C | 0.72 (0.88) | 0.59 (0.86) | 0.12 (-0.04 to 0.29) |
|  | ECG | 0.36 (0.63) | 0.22 (0.51) | 0.14 (0.03 to 0.24) |
|  | Diabetic retinopathy | 2.26 (1.11) | 2.16 (1.24) | 0.10 (-0.11 to 0.32) |
|  | Footcare | 0.01 (0.09) | 0.02 (0.15) | -0.01 (-0.04 to 0.01) |
|  | Sub-total | 3.35 (2.71) | 2.99 (2.76) | 0.35 (-0.16 to 0.86) |
| Medication | Diabetes mellitus | 0.24 (0.43) | 0.20 (0.40) | 0.04 (-0.04 to 0.11) |
|  | Coronary heart disease | 0.12 (0.47) | 0.09 (0.42) | 0.03 (-0.05 to 0.11) |
|  | Hypercholesteremia | 0.54 (1.16) | 0.57 (1.18) | -0.03 (-0.24 to 0.18) |
|  | Others (Gastritis, hypothyroidism, allergy, psychological problems) | 0.17 (0.80) | 0.21 (0.90) | -0.05 (-0.20 to 0.11) |
|  | Sub-total | 1.07 (2.86) | 1.07 (2.90) | -0.01 (-0.53 to 0.51) |
| Inpatient hospital visit | Number of admissions | 0.02 (0.14) | 0.01 (0.11) | 0.01 (-0.01 to 0.03) |
|  | Overnight stay | 0.03 (0.16) | 0.02 (0.13) | 0.01 (-0.02 to 0.04) |
|  | Length of stay (days) | 0.39 (4.73) | 0.07 (0.65) | 0.32 (-0.29 to 0.93) |
|  | Sub-total | 0.44 (5.03) | 0.10 (0.89) | 0.34 (-0.33 to 1.00) |
| Transportation | Travel times (hour) | 1.05 (1.28) | 1.06 (1.14) | -0.01 (-0.24 to 0.22) |
| Food items | Recommended fruits and vegetables | 2.53 (2.64) | 1.91 (2.73) | 0.62 (0.12 to 1.12) |

*CI: Confident intervals; SD: Standard deviation; CIs were obtained from 10,000 bootstrap resampling technique; HbA1c: Glycated haemoglobin; ECG: Electrocardiogram; HA: Health Assistant; CMA: Community Medical Assistant; ANM: Auxiliary Nurse Midwife*

**Supplementary table 3: Estimated cost (i.e., in both US $ and Nepali Rupees) per patient measures throughout the 6 months trial**

| **Measurements** | **Comparison*** | | | | |
| --- | --- | --- | --- | --- | --- |
|  | Intervention (n=238) | | Control (n=243) | | Mean differences in US $ (95% CI) |
|  | Mean (US $ (NRs)) | SD (US $ (NRs)) | Mean (US $ (NRs)) | SD (US $ (NRs)) |  |
| Direct medical costs |  |  |  |  |  |
| Medical consultation | 4.16 (522.02) | 6.28 (786.26) | 3.85 (481.48) | 7.05 (882.66) | 0.32 (-0.87 to 1.52) |
| Screening | 14.31 (1,791.19) | 6.12 (766.22) | 13.43 (1,681.63) | 5.88 (736.18) | 0.88 (-0.20 to 1.95) |
| Medication | 9.35 (1,170.57) | 17.73 (2219.80) | 3.68 (460.93) | 8.40 (1,051.61) | 5.67 (3.18 to 8.15) |
| Inpatient services | 2.80 (349.95) | 30.47 (3814.84) | 1.45 (181.54) | 4.68 (585.94) | 1.95 (-1.93 to 5.83) |
| Sub-total | 30.62 (3,833.62) | 60.60 (7,587.12) | 22.41 (2,805.73) | 26.01 (3,256.45) | 8.82 (0.18 to 17.45) |
| Direct non-medical costs |  |  |  |  |  |
| Transportation | 0.65 (81.08) | 0.77 (117.19) | 0.65 (81.08) | 0.68 (85.14) | -0.01 (-0.15 to 0.13) |
| Recommended Food items | 1.60 (200.41) | 2.18 (272.94) | 2.06 (258.30) | 2.11 (264.17) | -0.46 (-0.85 to -0.07) |
| Sub-total | 2.25 (281.70) | 2.95 (369.34) | 2.71 (339.29) | 2.79 (349.31) | -0.47 (-1.00 to 0.06) |
| Indirect costs |  |  |  |  |  |
| Patient income loss | 12.83 (1,606.21) | 24.88 (3114.98) | 9.11 (1,141.19) | 6.55 (820.06) | 3.71 (0.47 to 6.95) |
| Intervention cost |  |  |  |  |  |
| Phone call | 0.47 (59.20) | 0.08 (10.02) | na | na | na |
| Peer support | 2.52 (315.50) | 1.37 (171.52) | na | na | na |
| Intensive training | 14.33 (1,794.72) | 7.27 (910.20) | na | na | na |
| Sub-total | 17.32 (2168.62) | 8.60 (1076.72) | na | na | na |
| Total costs | 63.02 (7,890.05) | 59.74 (7479.45) | 34.23 (4,285.57) | 16.42 (2,055.78) | 28.79 (20.91 to 36.67) |

*na: not applicable; CI: Confident intervals; SD: Standard deviation; CIs were obtained from 10,000 bootstrap resampling technique; *Pooled value from multiple imputation; Pooled SD was calculated by computing the average over the standard deviations*

**Supplementary figure 2: Proportion of costing categories across intervention and control group**


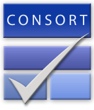
 Supplementary table 4: CONSORT 2010 checklist of information to include when reporting a randomised trial*

| Section/Topic | Item No | Checklist item | Reported on page No |
| --- | --- | --- | --- |
| Title and abstract | | | |
|  | 1a | Identification as a randomised trial in the title | 1 |
|  | 1b | Structured summary of trial design, methods, results, and conclusions (for specific guidance see CONSORT for abstracts) | 2-3 |
| Introduction | | | |
| Background and objectives | 2a | Scientific background and explanation of rationale | 3 |
|  | 2b | Specific objectives or hypotheses | 5 |
| Methods | | | |
| Trial design | 3a | Description of trial design (such as parallel, factorial) including allocation ratio | 5 |
|  | 3b | Important changes to methods after trial commencement (such as eligibility criteria), with reasons | 5 |
| Participants | 4a | Eligibility criteria for participants | 5 |
|  | 4b | Settings and locations where the data were collected | 5 |
| Interventions | 5 | The interventions for each group with sufficient details to allow replication, including how and when they were actually administered | 6 |
| Outcomes | 6a | Completely defined pre-specified primary and secondary outcome measures, including how and when they were assessed | 6-9 |
|  | 6b | Any changes to trial outcomes after the trial commenced, with reasons | 5-7 |
| Sample size | 7a | How sample size was determined | 5 |
|  | 7b | When applicable, explanation of any interim analyses and stopping guidelines | NA |
| Randomisation: |  |  |  |
| Sequence generation | 8a | Method used to generate the random allocation sequence | 5-7 |
|  | 8b | Type of randomisation; details of any restriction (such as blocking and block size) | 5 |
| Allocation concealment mechanism | 9 | Mechanism used to implement the random allocation sequence (such as sequentially numbered containers), describing any steps taken to conceal the sequence until interventions were assigned | NA |
| Implementation | 10 | Who generated the random allocation sequence, who enrolled participants, and who assigned participants to interventions | 5-7 |
| Blinding | 11a | If done, who was blinded after assignment to interventions (for example, participants, care providers, those assessing outcomes) and how | 5-7 |
|  | 11b | If relevant, description of the similarity of interventions | NA |
| Statistical methods | 12a | Statistical methods used to compare groups for primary and secondary outcomes | 10-11 |
|  | 12b | Methods for additional analyses, such as subgroup analyses and adjusted analyses | 11-12 |
| Results | | | |
| Participant flow (a diagram is strongly recommended) | 13a | For each group, the numbers of participants who were randomly assigned, received intended treatment, and were analysed for the primary outcome | 12 |
|  | 13b | For each group, losses and exclusions after randomisation, together with reasons | 12 |
| Recruitment | 14a | Dates defining the periods of recruitment and follow-up | 12 |
|  | 14b | Why the trial ended or was stopped | NA |
| Baseline data | 15 | A table showing baseline demographic and clinical characteristics for each group | 12 |
| Numbers analysed | 16 | For each group, number of participants (denominator) included in each analysis and whether the analysis was by original assigned groups | 13-16 |
| Outcomes and estimation | 17a | For each primary and secondary outcome, results for each group, and the estimated effect size and its precision (such as 95% confidence interval) | 13-16 |
|  | 17b | For binary outcomes, presentation of both absolute and relative effect sizes is recommended | NA |
| Ancillary analyses | 18 | Results of any other analyses performed, including subgroup analyses and adjusted analyses, distinguishing pre-specified from exploratory | 14-16 |
| Harms | 19 | All important harms or unintended effects in each group (for specific guidance see CONSORT for harms) | NA |
| Discussion | | | |
| Limitations | 20 | Trial limitations, addressing sources of potential bias, imprecision, and, if relevant, multiplicity of analyses | 16-19 |
| Generalisability | 21 | Generalisability (external validity, applicability) of the trial findings | 18-19 |
| Interpretation | 22 | Interpretation consistent with results, balancing benefits and harms, and considering other relevant evidence | 19 |
| Other information | | |  |
| Registration | 23 | Registration number and name of trial registry | 20 |
| Protocol | 24 | Where the full trial protocol can be accessed, if available | 5 |
| Funding | 25 | Sources of funding and other support (such as supply of drugs), role of funders | 21 |

*Citation: Schulz KF, Altman DG, Moher D, for the CONSORT Group. CONSORT 2010 Statement: updated guidelines for reporting parallel group randomised trials. BMC Medicine. 2010;8:18.*

**Graphical abstract**

1. **Histogram representing costs data**

1. **Histogram representing QALY data**

**Supplementary figure 3: Histogram representing data distribution (a) costs (b) QALY**

**Supplementary table 5: Modified park test outcomes (a) for costs (b) QALYs**

1. **Costs as a dependent variable**


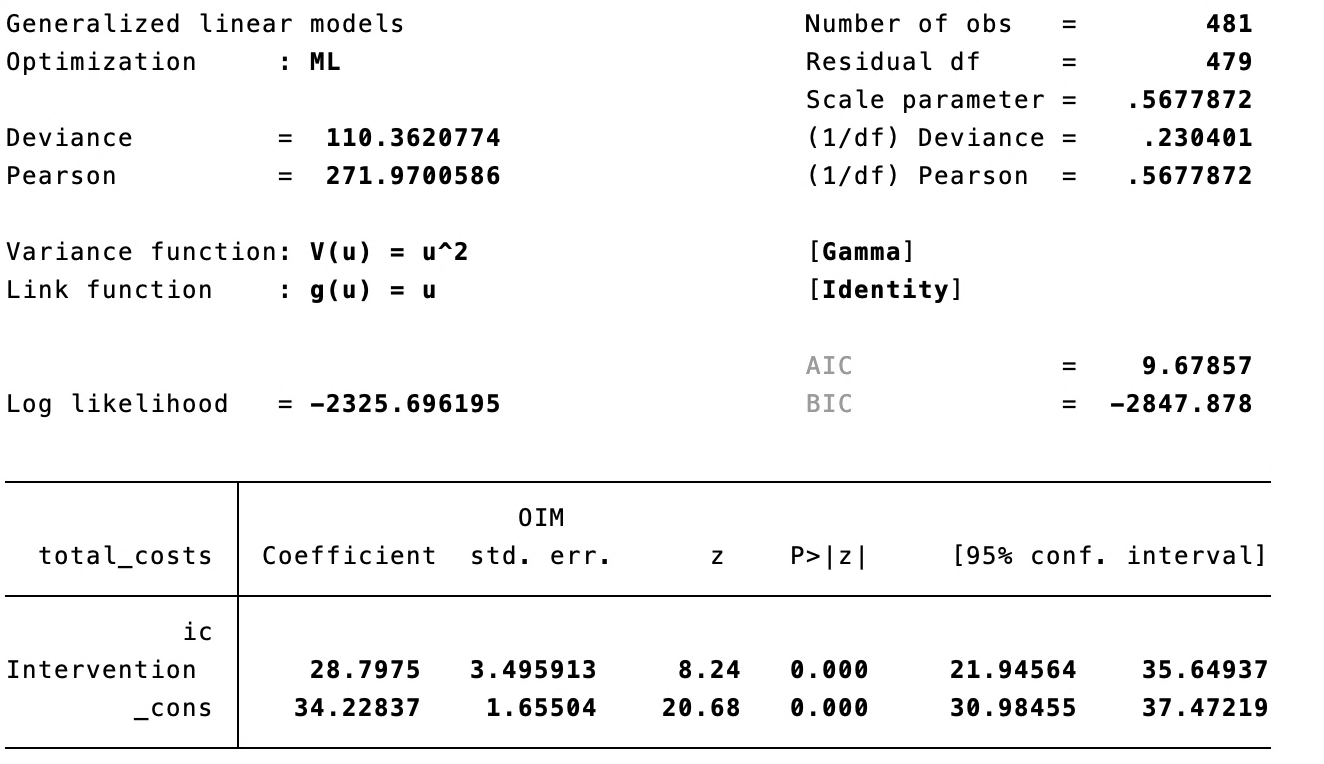



*Fit of a preliminary GLM Model for costs as a dependent variable*


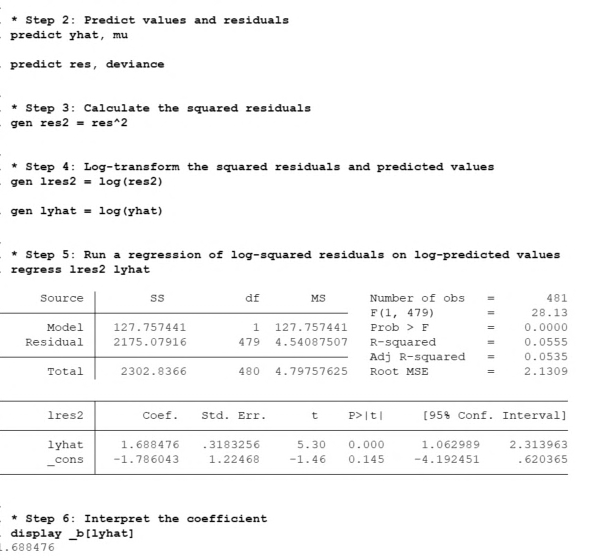


*‘Modified park test’ estimates for costs*

1. **QALYs as a dependent variable**


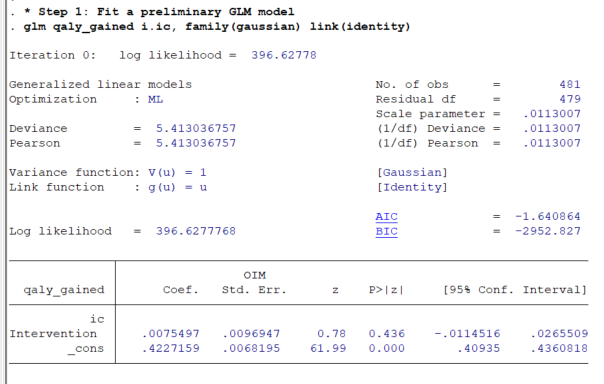


*Fit of a preliminary GLM Model for QALYs as a dependent variable*


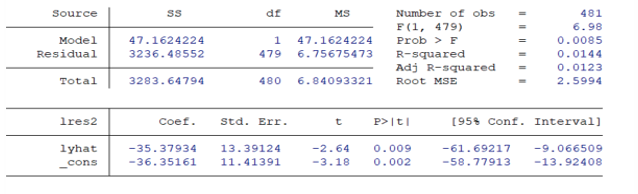


*‘Modified park test’ estimates for QALYs*

**Interpretation of beta for both costs and QALYs (the coefficient of lyhat) (1, 2).**

- If beta ≈ 0, the variance is constant, suggesting a Gaussian family.
- If beta ≈ 1, the variance is proportional to the mean, suggesting a Poisson family.
- If beta ≈ 2, the variance is proportional to the mean squared, indicating a Gamma family.
- If beta ≈ 3, the variance is proportional to the mean cubed, suggesting an Inverse Gaussian family.

**References**

1. Polgreen LA, Brooks JM. Estimating incremental costs with skew: a cautionary note. Appl Health Econ Health Policy. 2012;10(5):319-29.

2. Zhou J, Williams C, Keng MJ, Wu R, Mihaylova B. Estimating Costs Associated with Disease Model States Using Generalized Linear Models: A Tutorial. PharmacoEconomics. 2024;42(3):261-73.
